# Central Dental Association of Northern New Jersey

**Published:** 1895-02

**Authors:** 


### CENTRAL DENTAL ASSOCIATION OF NORTHERN NEW JERSEY.

### DISCUSSION OF MR. ALBERT E. WOOLF'S PAPER ON "AN ELECTRICAL DISINFECTANT."

(For Mr. Woolf's paper, see page 65.)

*Dr. Watkins.*—If this electrozone should be injected into a canal, so as to come in contact with the tissues at the end of the canal, would it act as an irritant?

*Mr. Woolf.*—I am glad that question has been asked, because it is a point that I did not touch upon. The doctor asks whether, if the disinfectant were to be injected into any of the canals so as to come in contact with the sensitive membrane lining it, it would act as an irritant. My answer is this, They have been making extended experiments in France with chlorine, and they find that, while chlorine acts as a germ-destroyer, it also acts as an irritant. I saw in last Sunday's *Herald* a report of some experiments made by saturating cotton with chlorine gas and applying it to discharging ulcers, and in the course of two or three weeks they found the ulcer much benefited. Now, the cure has been effected long before the two or three weeks elapsed, but it took two or three weeks for the inflammation that has been set up by the chlorine gas to subside; it took that long to get rid of the effects of the irritation set up by the chlorine. Now, this electrical disinfectant is not an irritant; on the contrary, when it is applied to the most sensitive sore it is soothing. For example, if you apply it to a cut you will never feel the cut until it is healed.

While constructing the plant at Quarantine during the cholera epidemic of 1893, one of the engineers had his arm scalded so badly that the flesh was removed from the wrist to the shoulder. We saturated a cloth with the disinfectant and applied it to the scalded arm, and within two days after the application the wound was ready to receive the salve for the formation of a new skin. Every particle of inflammation and irritation had been removed, which would prove beyond question that this electrical disinfectant is not an irritant. But in its application for very sore throats, if you use it too strong, you will find the membrane somewhat irritated. There is need of some caution there: after using it one should stay out of draught for fifteen or twenty minutes, because if a little inflammation should be set up it would be aggravated by draughts. I have not in a year seen one case where it has been injurious, or where it has not been beneficial.

I will state, also, in passing, that in one of the letters of Dr. Cohen, of Philadelphia, he writes, "Did I notify you that my daughter had aborted a stye on the eye with electrozone?" That is my experience also. I had a stye about six months ago. I put some diluted electrozone on the eyelid over night, and in the morning there was no sign of stye or swelling.

*Dr. Luckey.*—Where and when can we get samples of electrozone to try it?

*Mr. Woolf.*—I gave some prepared for medicinal use to Dr. Brown, and he tells me he has used it. I will send him another supply, and if you gentlemen will call on him he will let you have it.

*Dr. Luckey.*—Can you give me your address?

*Mr. Woolf.*—I can give you my address, but I am a very busy man, and it would take trouble off my shoulders if you would send to Dr. Brown. We have not started to make the disinfectant for medicinal uses,—that is, it is not on the market for sale,—but I will send to you and your friends all you require for experimental purposes; I will make up quite a little stock of it and send some of it to Dr. Brown.

*Dr. Luckey.*—I think this is a subject of such deep interest to us that I would like to know where we can obtain this electrozone for trial. If it proves as efficient in our work as it seems to have in other directions, it is the one thing that we have been waiting and longing for these many years, and that is the reason why I ask the question, When and where can we get it?

*Mr. Woolf.*—Dr. Brown will have all that you gentlemen can possibly use, and he will give you samples as fast as you want them for experimental purposes. I am sorry that I did not bring with me a copy of the *Dental Practitioner*, containing an article written by Dr. Bödecker—a gentleman whom I have never had the pleasure of meeting—on the benefits which his patients have received from the use of electrozone. In that article he cites different cases in which he used it, and states how he used it. I think it was written in the interests of your profession.

*Dr. Barlow.*—I understand this electrozone has been obtained at the S. S. White Dental Depot.

*Mr. Woolf.*—There is a possibility that that is so. Our agent has been out for some time, and he told me that this dental manufacturing company wanted some of the medicinal preparation, but I have been too busy to make it, and it is not on the market. I think the White Company has secured some that was manufactured for disinfecting purposes. I will say to those present that I feel myself to have been guilty of gross negligence in allowing that disinfectant to get on the market without having specially stated that it was not made for medicinal use. I feel that this has not been the fault of the S. S. White Dental Manufacturing Company.

*Dr. Sanger.*—What would be the effect of heat, as in a hot spray?

*Mr. Woolf.*—I think that if you were to heat it to boiling-point it would have an injurious effect. It is safe to heat it to blood-heat, but beyond that point there might be some chemical change,—just what, I do not know.

*Dr. Sanger.*—How would it act if used in a steam atomizer?

*Mr. Woolf.*—I think it is not necessary to use a steam atomizer. I think there are no objections to the use of the ordinary rubber atomizer.

*Dr. Sanger.*—Does it deteriorate by exposure to air?

*Mr. Woolf.*—But a trifle.

*Dr. Sanger.*—What is the effect of light?

*Mr. Woolf.*—I would always keep it away from bright light.

*Dr. Sanger.*—Would it have any effect on metal instruments, or be affected by them?

*Mr. Woolf.*—It would not affect instruments, unless they were left in it quite a little time.

*Dr. Sanger.*—Mr. President, I understand that Dr. Wright, of New York, has had some experience with electrozone. We would like to hear from him.

*Dr. Wright.*—I read the article of Dr. Bödecker, and have tried some of the suggestions he makes. I have used electrozone in the treatment of pericementitis and pyorrhœa alveolaris, and also in the treatment of dead teeth, and have been very successful. I should like to know what effect it would have if injected into an abscess.

*Dr. G. Carleton Brown.*—Mr. President, I believe Dr. Wilson has made some experiments with electrozone.

*The President.*—We would be pleased to hear from Dr. Wilson.

*Dr. Wilson.*—Mr. President, I have made some experiments, at the solicitation of Dr. Brown, not having any pecuniary interest in this electrozone, or any other interest except from a scientific stand-point. Dr. Brown called upon me two weeks ago and asked me if I would make some experiments with it. I told him I would be pleased to do so. I have found, in the first place, that it allayed the odor from putrefactive urine. I had some urine in the office for several days, and it became very offensive; I added a few drops of electrozone, and the odor entirely disappeared. I have also taken decomposed tissue and immersed it in this fluid, using it as a preservative fluid, and it preserved it perfectly. I have also made some bacteriological tests. I have here two culture-tubes. With this tube I took a solution of electrozone and swabbed out the interior of the tube, as well as the surface of the agar. Then I inoculated the tube with germs. I did the same with the other tube, not treating it with electrozone; then put it under similar circumstances,—favorable circumstances for propagation. That was last Friday afternoon. You see the germs, which have grown very rapidly in the tube in which electrozone was not used, while it has not appeared at all in the agar, in the tube in which the electrozone was used, showing that germs cannot exist in the presence of electrozone. This to me is a most practical test. While I am not yet satisfied with my experiments, I am glad to say that, so far as I know, this electrozone is a most perfect germicide.

*Dr. George C. Brown.*—Mr. President, since I have had this electrozone in my office I have made an experiment in a case of sensitive enamel. The teeth were so sensitive as to render it almost impossible to touch them without the patient responding as though it were an exposed pulp, or something of that kind, it was so painful. I have been battling with that sensitive enamel for years; the lady has been a patient of mine ever since I came to Elizabeth, twelve or fourteen years ago. Without exception, she has the most sensitive enamel of any person I have ever met. She has come to me time after time with, as she supposed, a cavity in the teeth, and wanted it filled, and upon examination I have found no sign of decay whatever. She has been unable to eat anything very sweet or very sour. Shortly after I received the electrozone from Mr. Woolf this lady was in my office, and I said to her, "I have something here which I would like you to try; I don't know whether it will have any effect in your case, but if you will be kind enough to take it home and try it I would be obliged to you;" and I gave her a bottle of electrozone. Three or four weeks after that I received this letter:

SUMNER PLACE, October 31, 1894.

DR. BROWN:

DEAR SIR,—After trying the electrified sea-water faithfully, I am glad to tell you that I am eating figs without any discomfort from sensitive teeth. My teeth were so sensitive before that I could hardly believe it when you said there was no decay. Where can I get more of this wonderful sea-water, as my supply is nearly gone? I hope it will be sold cheaply, as I shall want it in generous quantities as long as I live, or until I get false teeth. Please let me know where it can be had, and the price, and oblige.

This lady is the wife of one of the professors in Rutgers College. I had no idea when I gave her the preparation that it would have any effect; I gave it to her as an experiment, and I give you the result. I saw her husband on Thursday afternoon, and he said she was perfectly comfortable, and had no return of the trouble.

(Vice-President Richards takes the chair.)

*Dr. Sanger.*—Mr. President, I move you that a vote of thanks be tendered to Mr. Woolf for his kindness in coming here and presenting to us this very interesting subject.

Dr. Sanger's motion was carried.

*Dr. G. Carleton Brown.*—I believe there are a number of gentlemen present who have had samples of this electrozone and have experimented with it. I think Dr. Meeker said he has used it successfully in a case of perforated antrum.

*Dr. Meeker.*—Mr. President, through the courtesy of Dr. Brown, I was presented with a bottle of electrozone, and, after reading the article in the *Dental Practitioner*, written by Dr. Bödecker, I tried electrozone on some dead pulps,—cases where I removed the pulps and filled at once. I think I have performed about ten operations of that kind since I have had the electrozone, and, on the strength of Dr. Bödecker's article, I filled immediately, and there has been no subsequent trouble, although I was, of course, fearful of it in each case. Last week I had a case of perforation of the antrum under the twelfth-year superior molar. I enlarged the canal. The lady complained of pus dropping in her throat from the fauces at night when reclining in bed. I opened up from the centre, and found that the pulp was dead. I went to the end of the palatine root, finding considerable pus there. I took an abscess-syringe and syringed with electrozone, full strength, into the antrum. The lady complained of a little pain at the time. I stopped the cavity with bibulous paper and told her to come the next day. I did not see anything of her until Saturday. She then said she had not had any further trouble, and so thought it was not necessary to come to me. On Saturday I removed the bibulous paper and entered the antrum. I found spiculæ of bone there, and I concluded there was slight necrosis. The pus had stopped flowing entirely, and she had had no trouble with the throat. That was to me a very good test of the benefit of electrozone as a powerful antiseptic. I would not have dared to use five-per-cent. pyrozone in that way.

*Dr. Barlow.*—I received a bottle of electrozone, and I tried it in about the same manner as Dr. Meeker has stated. I have filled several teeth, after cleaning out the pulp-canals and using electrozone in them. Saturday afternoon a lady came to me, through a physician, bringing a child eight years of age, the child having a sixth-year molar which a dentist had attempted to remove two or three years ago, just after it erupted, and from that time up to the present there had been a discharge from the external part of the tooth. There has been a running abscess ever since. The child is still under the care of the physician, and he told her on Saturday that he did not know whether there was a root there causing the trouble or whether there was necrosis, and she had better come to a dentist in reference to it. I took a probe and entered it, and I thought I detected a root. I syringed it with electrozone, full strength, washing it out thoroughly two or three times, pumping the electrozone into it. Pus flowed out very freely. I told her to come back this morning. She came about nine o'clock, and she said, "Doctor, that child has not had so little pain for the past month as she has had since you injected that medicine." I told her

I thought there was a root there. I was quite certain there was part of a root left, and probably some necrosis. I made no further attempt; I did not want to take the case out of the doctor's hands, so I told her to report to him. She said the child had had less pain since Saturday afternoon, when I treated the abscess with electrozone.

*Dr. Sanger.*—Mr. President, I received a bottle of this electrozone, through the courtesy of Dr. Brown, about a week or ten days ago, and the first case I tried it on was one of those pulp-canals such as you are all familiar with; it filled the operating-room with its odor. I treated that canal, removing the pulp, and swabbing out the canal thoroughly. I then introduced a broach with clean cotton, and the only odor that I could detect was that of chlorine. The thought that came to my mind was chloride of lime; I did not think at first what it was. The purifying of the canal seemed to be perfect. I afterwards introduced the Evans root-dryer, hot, and I failed to detect the customary odor that comes when you first withdraw the hot dryer from the canal and put it into the flame. That led me to believe that that canal at least was thoroughly purified, and I filled at once, without any trouble so far. I am still experimenting with the remedy, and I expect great results.

*Vice-President Richards.*—Are there any others who wish to report on this subject? I believe quite a number of our members have received samples of electrozone from Dr. Brown.

*Dr. George C. Brown.*—If any of those present would like to try this remedy, and they will drop me a line, when I receive a supply from Mr. Woolf, I shall be happy to send them some.

*Dr. Wilson.*—I would like to say that, as regards its action upon mucous membranes, I have not had sufficient experience to speak very positively; but I am now experimenting upon some traumatic conditions of the nasal mucous membrane, and thus far it has worked beautifully. I do not believe, however, that this substance is a cure-all. There are certain conditions of the mucous membrane that it does not irritate. In some cases it acts as a stimulant.

*Mr. Woolf.*—Mr. President, I cannot refrain from expressing my thanks to those present for the patient attention they have given to my presentation of this subject and the courtesy they have shown me this evening, and I wish to publicly acknowledge my debt of gratitude.

*Dr. Meeker.*—Mr. President, I am glad to say that Mr. Woolf has just consented to read a paper before the State Society at the

August meeting. We expect at that meeting about eight hundred dentists, coming from all parts of the United States, and some from foreign countries. The New Jersey State Society and the National Association will meet in the same week at Asbury Park. The auditorium has been engaged for the occasion, and there will be ten chairs and thirty clinicians. It will be one of the grandest dental meetings ever held in this country.

*Vice-President Richards.*—Gentlemen, I have received a bottle of this electrozone, and I applied it to an aching tooth, not expecting any benefit, but the lady said the tooth did not ache after she went out of the office. It stopped the toothache immediately, and the lady was nearly frantic when she came in. You can take that for what it is worth.

*Dr. G. Carleton Brown.*—I want to speak of the action of this remedy in a case of a class which has not been touched upon here to-night. Two weeks ago a gentleman came in, suffering from periostitis. He had some bridge-work in his mouth, and the roots it was anchored to indicated violent irritation. I did not know exactly what to do, so I thought I would try electrozone. I saturated some cotton with it and put it on the roots, with the result that he left the house feeling perfectly comfortable; had no pain whatever. He is a writer on a paper in Syracuse, and he sent me a paper the other day with a long editorial on the wonderful effects of electrozone, so I suppose the cure was permanent.

